# Supplementary material for: Exploring associations between social media addiction, social media fatigue, fear of missing out and sleep quality among university students: A cross-section study
Source: PLoS One. 2023 Oct 5;18(10):e0292429. doi: 10.1371/journal.pone.0292429 (PMC10553250; doi:10.1371/journal.pone.0292429)
Supplement: S1 Table — (DOCX) [file pone.0292429.s002.docx]

S1 Table General characteristics and Internet use of the participants (n = 2744)

| Variable | n (%) | Variable | n (%) |
| --- | --- | --- | --- |
| **Gender** |  | **Number of social media accounts** |  |
| Female | 1899 (69.2) | 0~2 | 833 (30.4) |
| Male | 845 (30.8) | 3~4 | 1214 (44.2) |
| **Residence** |  | 5~6 | 436 (15.9) |
| City | 1199 (43.7) | 7~8 | 116 (4.2) |
| Rural | 1545 (56.3) | 9~ | 145 (5.3) |
| **Single child** | 1905 (69.4) | **Do you spend more time on social media than real world?** |  |
| **Education** |  | Less | 394 (14.4) |
| Junior college students | 1147 (41.8) | The same | 745 (27.2) |
| Undergraduate students and above | 1597 (58.2) | Slightly | 969 (35.3) |
| **Marital status of parents** |  | Much | 636 (23.2) |
| Married | 2410 (87.8) | **Time spent on social media per day (h)** |  |
| Single parent or stepparent | 284 (10.3) | 0~2 | 516 (18.8) |
| Others | 50 (1.8) | 2~4 | 1020 (37.2) |
| **Self-reported health status** |  | 4~6 | 743 (27.1) |
| Good | 2360 (86.0) | 6~8 | 267 (9.7) |
| Fair | 481 (17.5) | 8~ | 198 (7.2) |
| Bad | 28 (1.0) | **Browsing social media before bed** |  |
| Chronic disease | 38 (1.4) | Strongly disagree | 74 (2.7) |
| History of serious illness | 18 (0.7) | Disagree | 282 (10.3) |
| Family history | 11 (0.4) | Not agree | 927 (33.8) |
| **Purposes of using social media** |  | Agree | 813 (29.6) |
| To stay in touch with what my friends are doing | 2316 (84.4) | Strongly agree | 648 (23.6) |
| To research/find products to buy | 1754 (63.9) |  |  |
| To find funny or entertaining contents | 1787 (65.1) |  |  |
| Learning | 1777 (64.8) |  |  |
| To stay up-to-date with news and current events | 1707 (62.2) |  |  |
| Playing game | 1265 (46.1) |  |  |
| To share photos or videos with others | 952 (34.7) |  |  |
| To initiate a topic | 525 (19.1) |  |  |
| Because a lot of my friends are on them | 479 (17.5) |  |  |
| Others | 314 (11.4) |  |  |
