# Supplementary material for: Exploring associations between social media addiction, social media fatigue, fear of missing out and sleep quality among university students: A cross-section study
Source: PLoS One. 2023 Oct 5;18(10):e0292429. doi: 10.1371/journal.pone.0292429 (PMC10553250; doi:10.1371/journal.pone.0292429)
Supplement: Attachment — Submitted filename: Response to editor and reviewers.docx [file pone.0292429.s003.docx]

Dear. Ugurcan Sayili, M.D:

On behalf of my co-authors, I thank you very much for giving us an opportunity to revise our manuscript, we also deeply appreciate the editor and reviewers for their positive and constructive comments and suggestions on our manuscript entitled “Exploring associations between social media addiction, social media fatigue, fear of missing out and sleep quality among university students: a cross-section study” (ID: PONE-D-22-10183). Those comments are all valuable and very helpful for revising and improving our paper, as well as providing important guidance for our research.

We have studied editor and reviewers’ comments carefully and have made corresponding revisions that we hope to meet with qualifications for publication. We have tried our best to revise our manuscript according to the comments. Attached please find the revised version, which we would like to submit for your kind consideration. The major changes and revisions have been highlighted in red, which can be turned into black by rejecting all the changes. We hope that these revisions are satisfactory for your serious consideration to publish this manuscript in Plos one. We would like to express our appreciation again to you and reviewers for the comments on our paper.

Looking forward to hearing back from you.

Correspondence and phone calls about the paper should be directed to Xinhong Zhu at the following address, phone, and e-mail address:

Dr. Xinhong Zhu

School of Nursing, Hubei University of Chinese Medicine, 430061 16# Huangjiahu West Road, Wuhan, China, E-mail: zxh88@hbtcm.edu.cn

Thanks for your attention to our paper.

We are very grateful to the reviewers and editor for their comments and suggestions for revisions. The manuscript has been substantially improved according to the suggestions of reviewers and editor. The main corrections in the paper and the responses to the reviewer’s comments are addressed below.

Journal Requirements:

When submitting your revision, we need you to address these additional requirements.

1. Please ensure that your manuscript meets PLOS ONE's style requirements, including those for file naming. The PLOS ONE style templates can be found at

https://journals.plos.org/plosone/s/file?id=wjVg/PLOSOne_formatting_sample_main_body.pdf and https://journals.plos.org/plosone/s/file?id=ba62/PLOSOne_formatting_sample_title_authors_affiliations.pdf

Response: We really thank you for your careful review on our manuscript. We have downloaded MANUSCRIPT BODY FORMATTING GUIDELINES and TITLE, AUTHOR, AFFILIATIONS FORMATTING GUIDELINES and revised manuscript to meet PLOS ONE's style requirements.

2. We suggest you thoroughly copyedit your manuscript for language usage, spelling, and grammar. If you do not know anyone who can help you do this, you may wish to consider employing a professional scientific editing service.

Whilst you may use any professional scientific editing service of your choice, PLOS has partnered with both American Journal Experts (AJE) and Editage to provide discounted services to PLOS authors. Both organizations have experience helping authors meet PLOS guidelines and can provide language editing, translation, manuscript formatting, and figure formatting to ensure your manuscript meets our submission guidelines. To take advantage of our partnership with AJE, visit the AJE website (http://learn.aje.com/plos/) for a 15% discount off AJE services. To take advantage of our partnership with Editage, visit the Editage website (www.editage.com) and enter referral code PLOSEDIT for a 15% discount off Editage services. If the PLOS editorial team finds any language issues in text that either AJE or Editage has edited, the service provider will re-edit the text for free.

Upon resubmission, please provide the following:

The name of the colleague or the details of the professional service that edited your manuscript

A copy of your manuscript showing your changes by either highlighting them or using track changes (uploaded as a *supporting information* file)

A clean copy of the edited manuscript (uploaded as the new *manuscript* file).

Response: Thank you very much for your serious review and kind reminding. We invited Pro. Fen Yan to provide language editing.

Fen Yang: Nurse educator, Health Care Management, School of Nursing, Hubei University of Chinese Medicine,

So far, she has published 28 articles. Recent articles as corresponding author are as follows

1. Li, C., Wu, M., Qiao, G., Gao, X., Hu, T., Zhao, X., Zhu, X., & **Yang, F.** (2023). Effectiveness of Continuity of Care in Reducing Depression Symptoms in Elderly: A Systematic Review and Meta‐analysis. International Journal of Geriatric Psychiatry, e5894.
2. Hu, T., Zhao, X., Wu, M., Li, Z., Luo, L., Yang, C., & **Yang, F.** (2022). Prevalence of depression in older adults: A systematic review and meta-analysis. Psychiatry research, 114511.
3. Zhao, X., Hu, T., Qiao, G., Li, C., Wu, M., **Yang, F.,** & Zhou, J. (2022). Psychometric properties of the smartphone distraction scale in Chinese college students: validity, reliability and influencing factors. Frontiers in psychiatry, 13.
4. Li, C., **Yang, F.,** Yang, B.X. et al. Experiences and challenges faced by community mental health workers when providing care to people with mental illness: a qualitative study. BMC Psychiatry 22, 623 (2022).

3. We note that the grant information you provided in the ‘Funding Information’ and ‘Financial Disclosure’ sections do not match. When you resubmit, please ensure that you provide the correct grant numbers for the awards you received for your study in the ‘Funding Information’ section.

Response: Thank you very much for your serious review and kind reminding. We have modified grant numbers of funds, which was consist with “Financial Disclosure” in the manuscript.

4. In your Data Availability statement, you have not specified where the minimal data set underlying the results described in your manuscript can be found. PLOS defines a study's minimal data set as the underlying data used to reach the conclusions drawn in the manuscript and any additional data required to replicate the reported study findings in their entirety. All PLOS journals require that the minimal data set be made fully available. For more information about our data policy, please see http://journals.plos.org/plosone/s/data-availability.

Upon re-submitting your revised manuscript, please upload your study’s minimal underlying data set as either Supporting Information files or to a stable, public repository and include the relevant URLs, DOIs, or accession numbers within your revised cover letter. For a list of acceptable repositories, please see http://journals.plos.org/plosone/s/data-availability#loc-recommended-repositories. Any potentially identifying patient information must be fully anonymized.

Important: If there are ethical or legal restrictions to sharing your data publicly, please explain these restrictions in detail. Please see our guidelines for more information on what we consider unacceptable restrictions to publicly sharing data: http://journals.plos.org/plosone/s/data-availability#loc-unacceptable-data-access-restrictions. Note that it is not acceptable for the authors to be the sole named individuals responsible for ensuring data access.

We will update your Data Availability statement to reflect the information you provide in your cover letter.

Response: We sincerely thank for your suggestion. We modified the part of “Data Availability Statement”.

All relevant data are within the manuscript and its Supporting information file.

5. We note that you have indicated that data from this study are available upon request. PLOS only allows data to be available upon request if there are legal or ethical restrictions on sharing data publicly. For more information on unacceptable data access restrictions, please see http://journals.plos.org/plosone/s/data-availability#loc-unacceptable-data-access-restrictions.

In your revised cover letter, please address the following prompts:

a) If there are ethical or legal restrictions on sharing a de-identified data set, please explain them in detail (e.g., data contain potentially sensitive information, data are owned by a third-party organization, etc.) and who has imposed them (e.g., an ethics committee). Please also provide contact information for a data access committee, ethics committee, or other institutional body to which data requests may be sent.

b) If there are no restrictions, please upload the minimal anonymized data set necessary to replicate your study findings as either Supporting Information files or to a stable, public repository and provide us with the relevant URLs, DOIs, or accession numbers. For a list of acceptable repositories, please see <http://journals.plos.org/plosone/s/data-availability#loc-recommended-repositories>.

We will update your Data Availability statement on your behalf to reflect the information you provide.

Response: We sincerely thank for your suggestion. All relevant data are within the manuscript and its Supporting information file.

6. PLOS requires an ORCID iD for the corresponding author in Editorial Manager on papers submitted after December 6th, 2016. Please ensure that you have an ORCID iD and that it is validated in Editorial Manager. To do this, go to ‘Update my Information’ (in the upper left-hand corner of the main menu), and click on the Fetch/Validate link next to the ORCID field. This will take you to the ORCID site and allow you to create a new iD or authenticate a pre-existing iD in Editorial Manager. Please see the following video for instructions on linking an ORCID iD to your Editorial Manager account: <https://www.youtube.com/watch?v=_xcclfuvtxQ>.

Response: Great thanks to you for your careful review and valuable suggestion. ORCID iD of corresponding author is shown as followed.

Xinhong Zhu: https://orcid.org/0000-0001-7356-7401

Reviewers' comments:

Reviewer #1:

Comments on Exploring associations between social media addiction, social media fatigue, fear of missing out and sleep quality among university students: a cross-section study.

1. Paper summary

This paper mainly studied the associations between the phenomena of problems on social media and sleep quality based on Chinese university students.

2. comments

Comment 1: There are some problems which should be well solved before it is considered for publication.

First, relevant research background needs to be supplemented in Introduction. In this part, it is noted that the negative consequences of social media use, harmful effects of social media use, SMF, SMA and damaging relationship between social media use and sleep problems in paragraph 1 through 3 respectively. However, the summary or prospect of “little attention is on the combined impacts of adverse effects of excessive social media use” in line 80, consistency of the combined impacts didn’t be displayed in the following hypothesis and design.

Response: We sincerely thank Reviewer 1 for your kind and professional comments of our paper, which not only encourage us to improve our present manuscript, but also provide some useful ideas for our future studies. Accordingly, we have studied comments carefully. Point-by-point replies to Reviewer #1 are listed below. We modified the paragraph 3, and rewrote the relationship of social media use (e.g. FoMO, SMA, and SMF) and sleep quality, and added 9 new references into the text. Meanwhile, the hypothesis of this study was revised.

Paragraph 3-4:

Recently, studies concerning social media use have stressed growing problems among social media in terms of sleep disorders. The relationships of delayed sleep onset, bad quality of sleep and increased time spent on internet were reported in previous studies [27, 28]. Frequent social media use could predict both poor mental health and sleep outcomes in youth [10]. Evidence showed that the impact of negative affect on sleep quality was mediated by FoMO and smartphone addiction in the sample of Chinese university students [29]. Additionally, higher level of FoMO was associated with higher level of insomnia, which in turn was associated with poorer mental health in the college students [30]. When individuals were addicted to the Internet, the overall pooled odds ratio of experiencing sleep disturbance was 2.20 (95% CI: 1.77–2.74), and a significant reduce in sleep duration was observed [31]. Furthermore, there was higher rates of insomnia among those with higher levels of SMA among Norwegian university students [32]. Although the relationship between chronic fatigue and sleep quality was confirmed among university students [33], few surveys on association of SMF and sleep quality were conducted among Chines university students. Given FoMO and SMA acting as SMF drivers [34], we hypothesized that SMF was related to sleep quality.

University students frequently suffer from poor sleep [35, 36]. The prevalence of insomnia in university students worldwide has been found to be 18.5%, much higher than the 7.4% rate in the general population [37]. The overall pooled prevalence of sleep disturbances and those suffering from insomnia symptoms in Chinese university students are 25.7% (95% CI: 22.5 ~28.9%) and 23.6% (95% CI: 18.9~29.0%), respectively [38]. Considering that social media use has been linked to poor sleep outcomes, we hypothesized that there were relationships of sleep quality with FoMO, SMF, and SMA. Thus, this study was conducted among Chinese university students, and correlated variables were investigated in detail, such as social media use, FoMO, SMA, SMF, sleep disturbances and sleep duration.

① In Abstract. For poor sleep quality, OR of SMA, 1.409, conflicts with Table 1.

② In line 111, it is stated that model 3 had three groups, but only a high and a low group are described.

③ In line 118, participants were split into two subgroups using SMA, which included the number 2752, in contrast to the number of participants with the criteria of exclusion.

④ The description of the percentage of women and rural residents in line 164 conflicts with Table S1.

⑤ The statement in section 3.2 that "self-reported health status [not bad] (OR = 1.814)" conflicts with Table 1.

Response: We sincerely thank you for your kind and professional comments on our manuscript. We apologize for our carelessness. In this study, because grouping of respondents based upon the midpoints of the score ranges produces very unequal numbers in the two categories, we have modified the cut-off of SMA, SMF and FoMO, and used the median values as the cut-off. The median scores of SMA, SMF and FoMO were 84, 50 and 32, respectively.

Meanwhile, we have modified our mistakes as followed:

1. In the abstract, for poor sleep quality, the OR of SMA is 1.415, which is consistent with Table 1.

② The participants were divided into two groups using the median SMF score for statistical analysis: a high score group (≥50, n = 1374), and a low score group (<50, n = 1370).

③ The participants were divided into two groups using the median SMA score for statistical analysis: a high score group (≥84, n = 1376), and a low score group (<84, n = 1368).

④ Of the 2744 participants, 69.2% were female, 56.3% lived in rural and 69.4% were single-child, which are consistent with data shown in Table S1.

⑤ Self-reported health status [fair (OR = 1.795, 95% CI: 1.193~2.701), bad (OR = 3.474, 95%CI: 1.462~8.256)] affected sleep quality.

Thank you again for your comments on our manuscripts.

Reviewer #2: This research paper is well-structured and presents a thorough review of the literature. The analysis is clear and logical, and the conclusions are well-supported. The paper is well-written and easy to understand.

The findings of this study suggest that SMF, FoMO, and SMA are all significantly associated with sleep quality among university students. Specifically, higher levels of SMF, FoMO, and SMA were associated with decreased sleep quality. These results suggest that university students with higher levels of social media fatigue, fear of missing out, and social media addiction may be more likely to experience poorer sleep outcomes. These findings could have important implications for interventions that seek to improve the sleep outcomes of university students. It may be beneficial for such interventions to target these specific factors, such as SMF, FoMO, and SMA, to help improve sleep quality. Additionally, healthcare providers should be aware of the potential impact of these factors on university student sleep quality and consider addressing them in clinical care.

Comment 1: The research is relevant but the main problem is not up-to-date, and the sources need update and discussion. Mobile phone use during social media outage, nomophobia, and insomnia literature not been discussed which are all FOMO.

Response: We appreciate Reviewer #2 for warm work and positive suggestions earnestly, which are very helpful for improving our paper and future research. In the paragraph 3, we modified the hypothesis and added evidence for supporting the relationship of FoMO and sleep problems as followed.

Recently, studies concerning social media use have stressed growing problems among social media in terms of sleep disorders. The relationships of delayed sleep onset, bad quality of sleep and increased time spent on internet were reported in previous studies [26, 27]. Frequent social media use could predict both poor mental health and sleep outcomes in youth [10]. Evidence showed that the impact of negative affect on sleep quality was mediated by FoMO and smartphone addiction in the sample of Chinese university students [28]. Additionally, higher level of FoMO was associated with higher level of insomnia, which in turn was associated with poorer mental health in the college students [29]. When individuals were addicted to the Internet, the overall pooled odds ratio of experiencing sleep disturbance was 2.20 (95% CI: 1.77–2.74), and a significant reduce in sleep duration was observed [30]. Furthermore, there was higher rates of insomnia in those with higher levels of SMA among Norwegian university students [31]. Although the relationship between chronic fatigue and sleep quality was confirmed among university students [32], few surveys on association of SMF and sleep quality were conducted among Chinese university students. Given FoMO and SMA acting as SMF drivers [33], we hypothesized that SMF was related to sleep quality.

Meanwhile, we modified the part of limitation as followed:

Second, the study could not control for other factors, such as problematic social media use, nighttime social media use, weekend catch-up sleep, daytime nap, mobile phone use during social media outage, nomophobia and insomnia. Thus, future studies should include these variables.

Reviewer #3:

Comment 1: 1. It is appropriate for researchers to specify p values in a single format (with a lowercase p) throughout the article.

Response: Thank you very much for your serious review and kind reminding. We revised p values in a single format (with a lowercase p) in the part of methods and results.

Comment 2: 2. In the methodology section, the sleep duration categories should be corrected as “<7 h”, “7-8 h”, and “>8 h”.

Response: Great thanks to you for your careful review and valuable suggestion. In fact, if sleep duration categories were be corrected as “<7 h”, “7-8 h”, and “>8 h” in this study, the associations of sleep duration, SMA, SMF and FoMO were not observed.

Therefore, in order to explore relationship of sleep duration, SMA, SMF and FoMO, sleep duration was divided into six categories: “< 5 h”, “5-6 h”, “6-7 h”, “7-8 h”, “8-9 h”, and “>9 h” in this study. As shown in Table 2, compared to > 9 h duration, SMF was associated with an increased risk of 5-6 h sleep (OR = 2.226, 95%CI: 1.132~4.375), while the risk of 7-8 h sleep was OR = 1.296 (95%CI: 1.007~1.670). FoMO and SMA did not significantly affect sleep duration. As shown in Fig 1, the mean scores of SMF, SMA and FoMO were highest at 5-6 h sleep.

Table 2 Factors associated with sleep duration among university students

| Variables | < 5 h ^a^ | 5-6 h ^a^ | 6-7 h ^a^ | 7-8 h ^a^ | 8-9 h ^a^ |
| --- | --- | --- | --- | --- | --- |
|  | AOR (95%CI) | AOR (95%CI) | AOR (95%CI) | AOR (95%CI) | AOR (95%CI) |
| **SMF** | 0.984 (0.445~2.175) | 2.226 (1.132~4.375) * | 1.458 (1.061~2.002) * | 1.296 (1.007~1.670) * | 1.034 (0.814~1.314) |
| **SMA** | 1.848 (0.797~4.285) | 1.161 (0.588~2.294) | 1.110 (0.802~1.535) | 1.007 (0.778~1.305) | 1.046 (0.818~1.337) |
| **FoMO** | 1.007 (0.455~2.228) | 0.898 (0.473~1.703) | 1.020 (0.744~1.398) | 0.957 (0.744~1.232) | 0.994 (0.783~1.261) |

*: *p* < 0.05, ^a^: > 9 h as reference category

Fig 1 Mean score of SMF, SMA and FoMO across sleep duration.

Comment 3: 3. Logistic regression is used to identify predictors of dependent variables, if more than one independent predictor variable is evaluated, the p value in univariate analysis p<0.25* is tested using multivariate logistic regression analysis of clinically significant variables. A statement should be made that the variables observed in the text and in the table were tested using multivariate logistic regression analysis (* David Hosmer, Stanley Lemeshow, Rodney Sturdivant - Applied Logistic Regression-Wiley (2013), page 91).

Response: We deeply appreciate for your careful review on our manuscript. We modified the part of data analysis as followed:

Then, variables were entered with *p* < 0.25 into a multivariate logistic regression analysis to identify predictors of sleep duration in univariate analysis. Multivariate logistic regression analyses were conducted with short sleep duration as the outcome variable (> 9 h as the reference category), and social media use, SMF, SMA, FoMO, and demographic information as the exposure variables (Table 2).
