# Supplementary material for: Exploring associations between social media addiction, social media fatigue, fear of missing out and sleep quality among university students: A cross-section study
Source: PLoS One. 2023 Oct 5;18(10):e0292429. doi: 10.1371/journal.pone.0292429 (PMC10553250; doi:10.1371/journal.pone.0292429)
Supplement: Attachment — Submitted filename: Response to Reviewers.docx [file pone.0292429.s004.docx]

Dear. Ahsan Ali

On behalf of my co-authors, I thank you very much for giving us an opportunity to revise our manuscript, we also deeply appreciate the editor for your positive and constructive comments and suggestions on our manuscript entitled “Exploring associations between social media addiction, social media fatigue, fear of missing out and sleep quality among university students: a cross-section study” (ID: PONE-D-22-10183. R2). Those comments are all valuable and very helpful for revising and improving our paper, as well as providing important guidance for our research.

We have studied editor’s comments carefully and have made corresponding revisions that we hope to meet with qualifications for publication. We have tried our best to revise our manuscript according to the comments. Attached please find the revised version, which we would like to submit for your kind consideration. The major changes and revisions have been highlighted in red, which can be turned into black by rejecting all the changes. We hope that these revisions are satisfactory for your serious consideration to publish this manuscript in Plos One. We would like to express our appreciation again to you and reviewers for the comments on our paper.

Looking forward to hearing back from you.

Correspondence and phone calls about the paper should be directed to Xinhong Zhu at the following address, phone, and e-mail address:

Dr. Xinhong Zhu

School of Nursing, Hubei University of Chinese Medicine, 430061 16# Huangjiahu West Road, Wuhan, China, E-mail: zxh88@hbtcm.edu.cn

Thanks for your attention to our paper.

We are very grateful to the editor for your comments and suggestions for revisions. The manuscript has been substantially improved according to the suggestions of editor. The main corrections in the paper and the responses to editor’s comments are addressed below.

I am grateful to the authors for their efforts in addressing the comments of the reviewers. However, I would like to suggest a few points before the paper moves on to the next step:

Comment 1. I recommend that the authors revise the abstract. In the "Background" section, the authors mention that "Social media use has been linked to poor sleep outcomes in the cyber age among university students, but the mechanisms underlying this association are not yet well understood." It would be appropriate to specify which mechanisms the authors studied in this article rather than making a general statement about the relationships. Additionally, a clear description of the results is needed in the abstract. Please avoid ambiguous statements such as "Poor sleep quality was significantly with SMF." Please write clearly.

**Response:** We sincerely thank your kind and professional comments of our paper, which not only encourage us to improve our present manuscript, but also provide some useful ideas for our future studies. Accordingly, we have studied comments carefully. Point-by-point replies to editor are listed below. We had modified the part of abstract.

**Background:** Social media use has been linked to poor sleep outcomes in the cyber age among university students, but the association between negative consequences of social media use and sleep problems are not yet well understood. The present study investigated the relationships of social media usage, social media fatigue (SMF), fear of missing out (FoMO), social media addiction (SMA) and sleep quality among university students.

**Method:** Online survey was conducted with 2744 respondents, who completed questionnaires including the Pittsburgh Sleep Quality Index (PSQI), FoMO, SMF, SMA and questions regarding sleep duration, social media use, health status, and demographic information.

Result: 19.9% of respondents suffered from sleep disturbance. 15.6% of participants slept less than 5 h, and 21.6% of subjects slept more than 9 h. Sleep quality was positively associated with SMF (OR = 1.387, 95% CI: 1.103~1.743), and SMA (OR = 1.415, 95% CI: 1.118~1.791). The relationship between FoMO and sleep disturbance was not significant. Compared to > 9 h duration, SMF increased risk of 5-6 h sleep (OR = 2.226, 95%CI: 1.132~4.375), 6-7 h sleep (OR = 1.458, 95%CI: 1.061~2.002), and 7-8 h sleep (OR =1.296, 95%CI: 1.007~1.670). FoMO and SMA did not significantly affect sleep duration. In addition, SMA (OR = 3.775, 95%CI: 3.141~4.537), FoMO (OR = 3.301, 95%CI: 2.753~3.958), and sleep disorder (OR = 1.284, 95%CI: 1.006~1.638) positively affected SMF level.

**Conclusion:** On experiencing negative consequences of social media use, such as SMF and SMA, university students are likely to have sleep problems. Further research exploring the interventions for improving poor sleep and negative outcomes of social media use should be conducted.

Comment 2. Please clarify how this study contributes to the existing literature and what it offers for practice in the last paragraph of the introduction section. Please align this with the discussion section as well.

**Response:** Thank you very much for your serious review and kind reminding. We had modified the last paragraph of the introduction section.

Although social media use has been linked to poor sleep outcomes, little research has focused on the relationship of FoMO, SMA, SMF and sleep quality. It is important to clarify the relationships of these four factors in order to detect the role of negative outcomes of social media use on sleep quality, and develop interventions to improve university students’ sleep quality for further study. Thus, this study was aimed to investigated the relationship between social media use, FoMO, SMA, SMF, sleep disturbances and sleep duration among Chinese university students.

Comment 3. Furthermore, I suggest that the authors include another heading after the introduction to provide an explanation of the relationship they have studied in this research and why these relationships are proposed. Also explain the theoretical foundation and practical evidence of the proposed relationships.

**Response:** Great thanks to you for your careful review and valuable suggestion. We had added another heading “Literature review and hypotheses” after the introduction and revised paragraph 3 and 4 as followed.

Recently, growing attention was paid on relationship of sleep disorders and social media usage. University students frequently suffer from poor sleep [26, 27]. The prevalence of insomnia in university students worldwide has been found to be 18.5%, much higher than the 7.4% rate in the general population [28]. The overall pooled prevalence of sleep disturbances and those suffering from insomnia symptoms in Chinese university students are 25.7% (95% CI: 22.5 ~28.9%) and 23.6% (95% CI: 18.9~29.0%), respectively [29]. The mechanisms behind the relationships between use of social media and sleep problems are not well established, but a theoretical model of the relationship has been proposed [30], suggesting several possible mechanisms. According to this model, social media use directly affect sleep quality by excess time consuming, or it may interfere with sleep by psychological arousal through the stimulating online content [30]. The relationships of delayed sleep onset, bad quality of sleep and increased time spent on internet were reported in previous studies [31, 32]. Frequent social media use could predict both poor mental health and bad sleep outcomes in youth [10]. Evidence showed that the impact of negative affect on sleep quality was mediated by FoMO and smartphone addiction in the sample of Chinese university students [33]. Additionally, insomnia partially mediated significant associations of interpersonal stress and FoMO with mental health in the college students [34]. When individuals were addicted to the Internet, the overall pooled odds ratio of experiencing sleep disturbance was 2.20 (95% CI: 1.77–2.74), and a significant reduce in sleep duration was observed [35]. Furthermore, there was higher rate of insomnia in those with higher level of SMA among Norwegian university students [36]. In summary, FoMO and SMA are closely with sleep quality. Although the relationship between chronic fatigue and sleep quality was confirmed among university students [37], few surveys on association of SMF and sleep quality were conducted among Chinese university students. Given FoMO and SMA acting as SMF drivers [38], we hypothesized that SMF was related to sleep quality.

Although social media use is linked with poor sleep outcomes, little research has focused on the relationship of FoMO, SMA, SMF and sleep quality. It is important to clarify the relationships of these four factors in order to detect the role of negative outcomes of social media use on sleep quality, and develop interventions to improve university students’ sleep quality for further study. Thus, this study was aimed to investigate the relationship between social media use, FoMO, SMA, SMF, sleep disturbance and sleep duration among Chinese university students.

Comment 4. Lastly, I appreciate the language improvement, but there are still a number of grammatical issues along with unclear expressions. Therefore, I recommend that the authors use the services of a professional copy-editor to help with this issue.

**Response:** We deeply appreciate for your careful review on our manuscript. We invited professor to improve the grammatical issues.
