# Supplementary material for: Exploring associations between social media addiction, social media fatigue, fear of missing out and sleep quality among university students: A cross-section study
Source: PLoS One. 2023 Oct 5;18(10):e0292429. doi: 10.1371/journal.pone.0292429 (PMC10553250; doi:10.1371/journal.pone.0292429)
Supplement: Attachment — Submitted filename: Response to eidtor.docx [file pone.0292429.s005.docx]

Dear. Ahsan Ali:

On behalf of my co-authors, I thank you very much for giving us an opportunity to revise our manuscript, we also deeply appreciate the editor for your positive and constructive comments and suggestions on our manuscript entitled “Exploring associations between social media addiction, social media fatigue, fear of missing out and sleep quality among university students: a cross-section study” (ID: PONE-D-22-10183.R3). Those comments are all valuable and very helpful for revising and improving our paper, as well as providing important guidance for our research.

We have studied editor’s comments carefully and have made corresponding revisions that we hope to meet with qualifications for publication. We have tried our best to revise our manuscript according to the comments. Attached please find the revised version, which we would like to submit for your kind consideration. The major changes and revisions have been highlighted in red, which can be turned into black by rejecting all the changes. We hope that these revisions are satisfactory for your serious consideration to publish this manuscript in the PLOS ONE. We would like to express our appreciation again to you and reviewer for the comments on our paper.

Looking forward to hearing back from you.

Correspondence and phone calls about the paper should be directed to Xinhong Zhu at the following address, phone, and e-mail address:

Dr. Xinhong Zhu

We are very grateful to your comments and suggestions for revisions. The manuscript has been substantially improved according to the suggestions of the editor. The main corrections in the paper and the responses to the editor’s comments are addressed below.

Comment 1

I appreciate your submission of the manuscript titled "Exploring associations between social media addiction, social media fatigue, fear of missing out, and sleep quality among university students: a cross-sectional study" to Plos One. After a rigorous evaluation conducted by multiple experts in the field, I have made a decision regarding the publication of your manuscript.

While your work addresses an interesting and significant topic, and the reviewers have noted substantial improvements in the paper, there remains a concern regarding language and grammar issues. To ensure the highest quality of publication, I strongly recommend that the authors consider seeking professional copy editing services to address these language and grammar concerns. Consequently, I invite you to revise and resubmit the paper after addressing these concerns.

Response: We deeply appreciate your comments. We have paid for a professional English editing service in AJE, and improved the quality of written English, including spelling mistakes, sentence structure and verb tense.
